# Hydrogen-Bonding-Driven Nontraditional Photoluminescence of a *β*-Enamino Ester

**DOI:** 10.3390/molecules28165950

**Published:** 2023-08-08

**Authors:** Wendi Xie, Junwen Deng, Yunhao Bai, Jinsheng Xiao, Huiliang Wang

**Affiliations:** Beijing Key Laboratory of Energy Conversion and Storage Materials, College of Chemistry, Beijing Normal University, Beijing 100875, China; 202221150112@mail.bnu.edu.cn (W.X.);

**Keywords:** hydrogen bond, nontraditional luminogens, through-space conjugation, *β*-enamino ester

## Abstract

Nontraditional luminogens (NTLs) do not contain any conventional chromophores (large π-conjugated structures), but they do show intrinsic photoluminescence. To achieve photoluminescence from NTLs, it is necessary to increase the extent of through-space conjugation (TSC) and suppress nonradiative decay. Incorporating strong physical interactions such as hydrogen bonding is an effective strategy to achieve this. In this work, we carried out comparative studies on the photoluminescence behaviors of two *β*-enamino esters with similar chemical structures, namely methyl 3-aminocrotonate (MAC) and methyl (*E*)-3-(1-pyrrolidinyl)-2-butenoate (MPB). MAC crystal emits blue fluorescence under UV irradiation. The critical cluster concentration of MAC in ethanol solutions was determined by studying the relationship between the photoluminescence intensity (UV–visible absorbance) and concentration. Furthermore, MAC exhibits solvatochromism, and its emission wavelength redshifts as the solvent polarity increases. On the contrary, MPB is non-emissive in both solid state and solutions. Crystal structures and theoretical calculation prove that strong inter- and intramolecular hydrogen bonds lead to the formation of large amounts of TSC of MAC molecules in aggregated states. No hydrogen bonds and thus no effective TSC can be formed between or within MPB molecules, and this is the reason for its non-emissive nature. This work provides a deeper understanding of how hydrogen bonding contributes to the luminescence of NTLs.

## 1. Introduction

Organic photoluminescent materials play a critical role in many areas of modern technology and have a significant impact on our daily lives [1,2]. Most traditional luminogens require structures with large through-bond conjugation (TBC), such as aromatic rings to originate photoluminescence [3,4]. The overlapped π orbitals and extended delocalization of electrons lead to a reduction in the highest occupied molecular orbital (HOMO)-lowest unoccupied molecular orbital (LUMO) energy gap. Recently, however, more and more nontraditional luminogens (NTLs) (also called clusteroluminogens) without large π-conjugated structures have been reported [5,6,7,8,9,10,11,12,13]. These NTLs contain electron-rich atoms such as N, O, S, and P, and/or unsaturated bonds such as C=O, C=C, C=N, and C≡N as nonconventional chromophores (NCCs). Many mechanisms have been reported to explain their luminescence, and among them, clusterization-triggered emission (CTE) [14] and through-space interaction [15,16,17,18,19] have been widely recognized. In the aggregated state of NTLs, NCCs clusterize under proper molecular conformation. Within the clusters, the electron clouds of NCCs overlap to form through-space conjugation (TSC), reducing the HOMO-LUMO energy gap, and hence resulting in the photoluminescence of NTLs [20,21,22,23].

To achieve photoluminescence from NTLs, it is necessary to increase the extent of TSC and to suppress nonradiative decay. Incorporating strong physical interactions such as hydrogen bonding is an effective strategy to achieve this [24]. Hydrogen bonding, which can promote electron delocalization [25], has been reported to enhance TSC in many previous works. Tang and colleagues reported a series of nontraditional luminescent polymers containing the amide group [26]. Hydrogen bonds shorten the distance between amide groups, facilitating their communication. Yan’s group prepared a hyperbranched polysiloxane with multicolor luminescence, in which hydrogen bonding promoted coplanar multiring TSC [27]. Guo’s group synthesized a nonconventional luminescence polymer with zwitterionic components [28]. The strong ionic and hydrogen bonds both help NCCs to form new emissive clusters. Our group reported the luminescence behavior of hexanal oxime, which aggregates and forms TSC through intermolecular hydrogen bonding [29]. We also fabricated a multi-color fluorescent polyacrylamide/poly(itaconic acid) hydrogel with tunable fluorescence [30]. Hydrogen bonding between the polymer chains led to the formation of clusters with rigidified conformations and more extended conjugation. Yuan’s group reported a series of aliphatic cyclic imides exhibiting room temperature phosphorescence stemming from molecular clustering and halogen effects. C=O···H−N hydrogen bonds accompanied by C=O···H−C, C=O···C=O, C=O···N−H, and C=O···X (Br and I) interactions are found in the single crystals of these molecules [31]. All of these interactions contribute to conformation rigidification.

However, no detailed and in-depth works have been carried out to understand how and to what extent hydrogen bonding affects TSC and, hence, the photoluminescence of NTLs. In this work, we carried out an investigation on this issue through comparative studies on the photoluminescence behaviors of two *β*-enamino esters with similar chemical structures, namely methyl 3-aminocrotonate and methyl (*E*)-3-(1-pyrrolidinyl)-2-butenoate, and the interactions in their aggregated structures. MAC crystal emits blue fluorescence under UV irradiation and MAC shows concentration-dependent and excitation-dependent fluorescence emissions. The critical cluster concentration (CCC) of MAC in ethanol solutions was determined by studying the relationship between the photoluminescence intensity (UV−visible absorbance) and concentration. Furthermore, MAC exhibited solvatochromism, and its emission wavelength redshifted as the solvent polarity increased. On the contrary, MPB is non-emissive in both solid state and solutions. Crystal structures and theoretical calculations prove that the H−N−C=C−C=O groups in MAC were in a planar conformation, and strong inter- and intramolecular hydrogen bonds led to the formation of large extents of TSC of MAC molecules in aggregated states. No hydrogen bonds and, hence, no effective TSC could be formed between or within MPB molecules, which is the reason for its non-emissive nature.

## 2. Results and Discussion

### 2.1. Photophysical Properties

**Figure 1:**
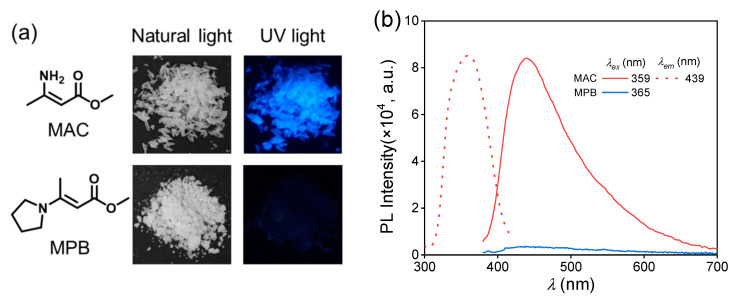
(**a**) Chemical structures of methyl 3-aminocrotonate (MAC) and methyl (*E*)-3-(1-pyrrolidinyl)-2-butenoate (MPB) and their photographs under the irradiation of natural light and 365 nm UV light. (**b**) Photoluminescent excitation (dashed line) and emission (solid line) spectra of MAC and MPB crystal.

The purchased methyl 3-aminocrotonate and synthesized methyl (*E*)-3-(1-pyrrolidinyl)-2-butenoate were recrystallized and then characterized by nuclear magnetic resonance (NMR) (Figures S1 and S2). Then, their photoluminescence behaviors were studied. The MAC crystal emitted a blue light under an irradiation of 365 nm UV light (Figure 1a), and its photoluminescence spectra are shown in Figure 1b. The maximum emission wavelength ($\lambda_{em}^{max}$) was 439 nm under the maximum excitation wavelength ($\lambda_{ex}^{max}$) of 359 nm with the fluorescence quantum yield (QY) of 4.5%. The emission lifetime of the MAC crystal was 2.18 ns (Figure S3), proving the photoluminescence (PL) emission was fluorescence. In comparison, MPB was nearly non-emissive with the naked eye. The photoluminescence of MPB was much weaker than MAC (Figure 1b), and its QY could not be obtained. So, MPB was considered as non-emissive.

**Figure 2:**
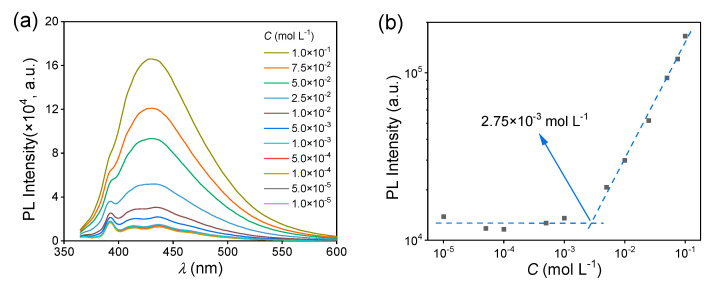

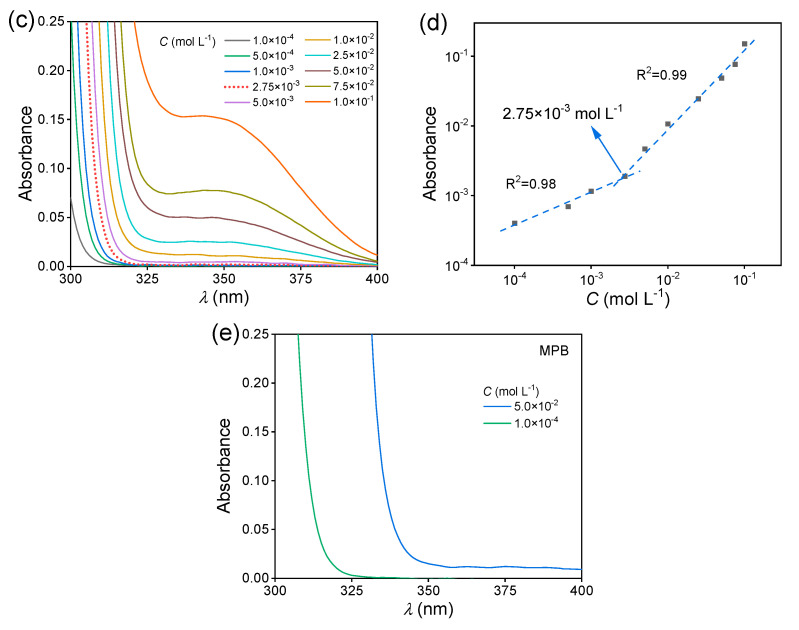
(**a**) Fluorescence emission spectra of MAC ethanol solutions with different concentrations ($\lambda_{ex}$ = 351 nm). (**b**) The double logarithmic plots of the PL intensity ($\lambda_{em}$ = 430 nm) vs. concentration of MAC ethanol solution. (**c**) UV−VIS absorption spectra of MAC ethanol solutions with different concentrations (300–400 nm). (**d**) The double logarithmic plots of absorbance (*λ* = 350 nm) vs. concentration of MAC ethanol solution. (**e**) UV−VIS absorption spectra of MPB ethanol solutions with different concentrations (300–400 nm).

Then, the fluorescence behavior of the MAC solutions was studied. The fluorescence intensity of the MAC ethanol solution increased with the increased concentration (Figure 2a), with a critical cluster concentration [32] of 2.75 × 10^−3^ mol L^−1^ (Figure 2b). When the concentration was lower than the CCC, the clusters were yet not completely formed and thus the PL intensity hardly changed. Meanwhile, above the CCC, clusters were formed and their number increased with the increased concentration, leading to the increase in fluorescence intensity. The UV−visible (UV−VIS) spectra of MAC ethanol solutions with different concentrations (Figure 2c and Figure S4) further prove the CCC. The shoulder peak at about 350 nm was attributed to the clusters. In the first stage below 2.75 × 10^−3^ mol L^−1^, the absorbance grew slower than in the second stage (Figure 2d). For comparison, MPB was still non-emissive in solutions (Figure S5). As shown in the UV−VIS spectra (Figure 2e and Figure S6), when the concentrations were both 5.0 × 10^−2^ mol L^−1^, MPB had a lower absorption than MAC over 350 nm, which means that the extent of aggregation of MPB was lower than that of MAC.

**Figure 3:**
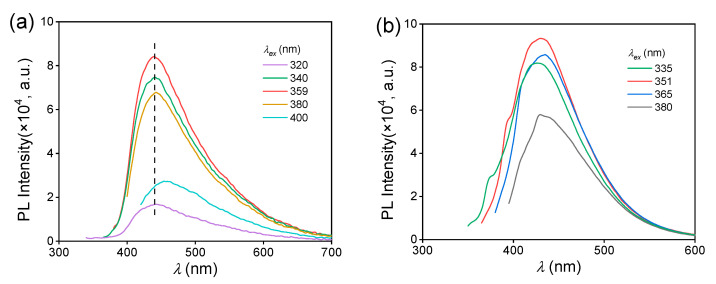
Fluorescence emission spectra of the crystal (**a**) and ethanol solution (**b**) of MAC under different $\lambda_{ex}$, *C* = 5.0 × 10^−2^ mol L^−1^.

To understand the difference in aggregated state between the MAC crystal and solution, their fluorescence behaviors were compared. For 5.0 × 10^−2^ mol L^−1^ ethanol solution, the $\lambda_{em}^{max}$ is 430 nm, which is shorter than that of MAC crystal (439 nm). The solution had a fluorescence QY of 1.7%, which was also lower than that of the crystal (4.5%). Furthermore, the $\lambda_{em}$ of the MAC crystal remained constant within the $\lambda_{ex}$ range of 320–380 nm (Figure 3a), while the $\lambda_{em}$ of the solution changed with increasing the $\lambda_{ex}$ (Figure 3b), exhibiting a more significant excitation-dependent emission (EDE).

**Figure 4:**
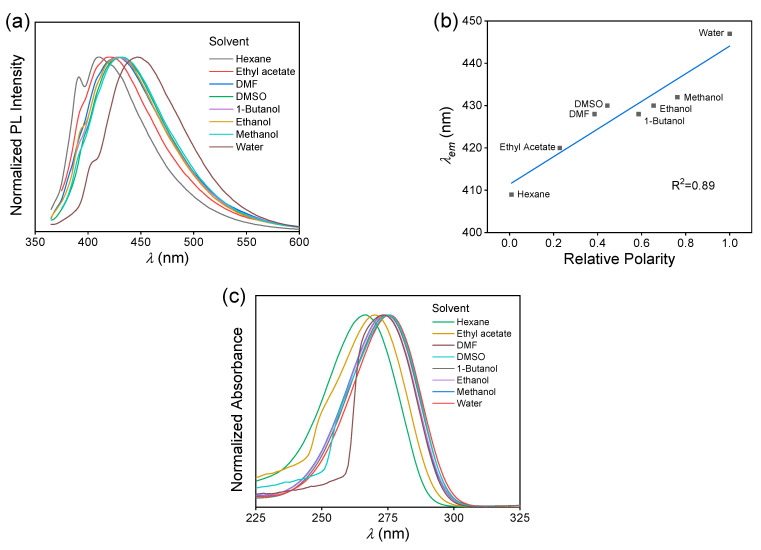
(**a**) Normalized fluorescence emission spectra of MAC in different solvents, *C* = 5.0 × 10^−2^ mol L^−1^. (**b**) Plots of $\lambda_{em}^{max}$ vs. relative polarity of the solvent of MAC solutions, *C* = 5.0 × 10^−2^ mol L^−1^. (**c**) Normalized UV−VIS absorption spectra of MAC in different solvents, *C* = 1.0 × 10^−4^ mol L^−1^.

The solvatochromism of MAC was studied by comparing the fluorescence behaviors of eight solutions. The solvents with polarity from low to high were hexane, ethyl acetate, *N*,*N*-dimethylformamide (DMF), dimethyl sulfoxide (DMSO), 1-butanol, ethanol, methanol, and water [33]. According to the fluorescence spectra of different solutions (Figure S7), the $\lambda_{em}^{max}$ exhibited a redshifted trend with increasing polarity, Δ*λ* = 38 nm, from 409 nm of hexane solution to 447 nm of aqueous solution (Figure 4a). It exhibited a positive linear correlation with the relative polarity, with an R^2^ of 0.89 (Figure 4b). The UV−VIS absorption spectra of these MAC solutions were measured (Figure S8 and Figure 4c). The maximum absorption wavelength measured at 1.0 × 10^−4^ mol L^−1^ also redshifted, but more slightly, with Δ*λ* = 10 nm.

### 2.2. Single Crystal Structure Analysis and Theoretical Calculation

**Figure 5:**
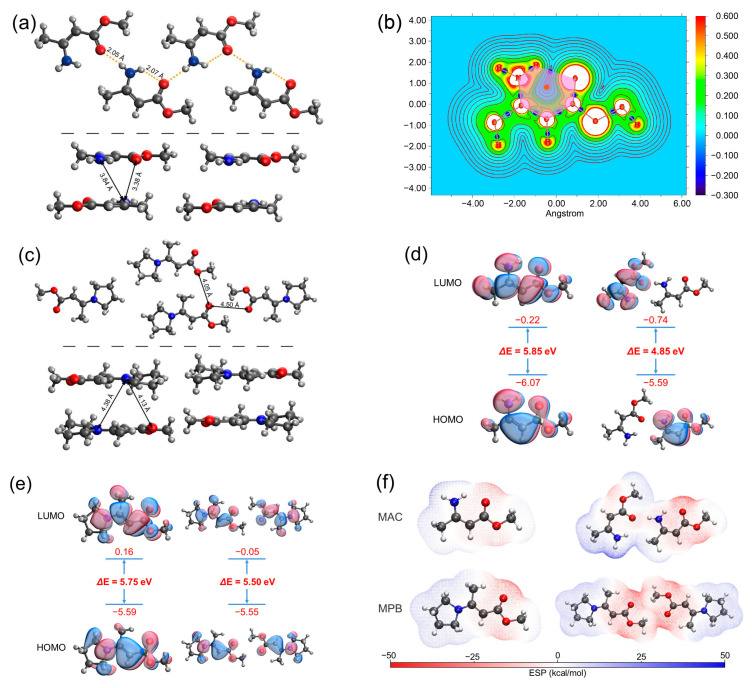
(**a**) Single crystal structure of MAC. The top part shows the intralayer structure and the bottom part shows the interlayer structure. (**b**) AIM analysis of MAC. The pink hexagon represents the six-membered ring, the orange dot represents the RCP, and the blue dots represent the BCPs. (**c**) Single crystal structure of MPB. (**d**,**e**) HOMO and LUMO electron densities of the selected monomers and dimers of MAC (**d**) and MPB (**e**). (**f**) Electrostatic potential (ESP) distribution of monomers and dimers of MAC and MPB.

The single crystal structure of MAC [34] is shown in Figure 5a. The H−N−C=C−C=O groups in MAC were in a planar conformation. The distance between H of –NH_2_ and O of C=O within the same molecule was 2.07 Å, and the distance between the H of –NH_2_ and O of C=O of the adjacent molecule within the same layer was 2.05 Å. Such short distances prove the existence of hydrogen bonds. While the distances of atoms of different layers were longer, for example, the shortest distance between N and O of different layers was 3.38 Å, and the shortest distance between N and N of different layers was 3.84 Å, which was too long for noncovalent interactions. Atoms in molecules (AIM) were employed to analyze the topology of the electron density. According to the AIM results, a bond critical point (BCP) was present between H of –NH_2_ and O of C=O, and a ring critical point (RCP) located at the center of the hexagon consisted of H−N−C=C−C=O, where the electron density gradient was zero, proving the formation of the quasi-six-membered ring containing intramolecular hydrogen bonding (Figure 5b). On the other hand, in the single crystal of MPB (Figure 5c), the N atom was restricted by the pyrrolidine ring, prohibiting the formation of hydrogen bonding. The distance between O of C=O and O of C=O of the adjacent molecule was 4.50 Å, and the distance between O of C=O and O of –OCH_3_ of the adjacent molecule was 4.05 Å, which were too far for the electron clouds to overlap. Besides, the distances of the atoms of different layers were also long; for example, the shortest distance between the N and O of different layers was 4.13 Å, and the shortest distance between the N and N of different layers was 4.38 Å, which were also too long for noncovalent interactions.

Theoretical calculations were performed to investigate the mechanism of fluorescence of MAC. We optimized the excited-state structures and simulated the frontier molecular orbitals of MAC and MPB single molecules and two adjacent molecules within the same layer by employing the PBE0 density functional theory [35] at the 6-311G** [36,37,38] level. The HOMO-LUMO energy gap of the MAC single molecule was 5.85 eV, and it shrank significantly to 4.85 eV in the dimer (Figure 5d), while the HOMO-LUMO energy gap of MPB shrank less, from 5.75 eV of a single molecule to 5.50 eV of two adjacent molecules (Figure 5e). Moreover, the HOMO and LUMO of the MAC dimer were each distributed over two molecules, indicating the through-space charge transfer (TSCT) between them. In contrast, the HOMO and LUMO of two adjacent MPB molecules were uniformly distributed over two molecules, indicating the negligible TSCT between them. The calculated energy of the intermolecular interactions between the adjacent molecules of MAC was 637.617 kJ mol^−1^, stronger than that of MPB (505.616 kJ^−1^). Furthermore, the electrostatic potential was calculated to show the distribution of electron clouds (Figure 5f). In the MAC single molecule, the negative charge was mainly located at the oxygen atoms and the positive charge was distributed around the amino group. The electron cloud of the amino group and oxygen atom of the carbonyl group were partially neutralized, which happened within both single molecule and dimer. In the MPB single molecule, the negative charge was mainly located at the oxygen atoms, and the positive charge was distributed around the pyrrolidine ring. Because of *E* configuration, little charge neutralization happened within a single molecule. The adjacent parts between two molecules had the same charge, also leading to little neutralization. Both the HOMO-LUMO energy gap and electrostatic potential distribution proved that large TSC formed in the MAC crystal and little TSC formed in the MPB crystal.

Based on the above single crystal structure analysis and theoretical calculation, the fluorescence mechanism of MAC crystal can be explained as follows. On the one hand, MAC and MPB had similar chemical structures and a similar extent of through-bond conjugation (TBC), but the latter was non-emissive, proving the minor contribution of TBC to the fluorescence of MAC. On the other hand, MAC formed a large TSC with the help of hydrogen bonding, while MPB could not form TSC effectively because of the absence of hydrogen bonding or other strong noncovalent interactions. Large TSC of MAC reduced its HOMO-LUMO energy gap, and hydrogen bonding stiffened the conformation and thus suppressed nonradiative decay, so the MAC was fluorescent.

In order to understand the luminescence behavior of MAC solutions, the S_0_ conformations of the MAC monomer and dimer in hexane and water were optimized (Figure S9). The monomer represents the isolated state and the dimer represents the aggregated state. No matter whether it was a monomer or dimer, the H−N−C=C−C=O groups in MAC maintained a planar conformation with the help of the intramolecular hydrogen bond. Furthermore, the S_0_-S_1_ energy gaps were calculated (Table 1). The horizontal comparison between the monomer and dimer showed a significant decrease in S_0_-S_1_ of the dimer (5.179 to 4.514 eV in hexane and 5.159 to 4.477 eV in water), proving the formation of TSC between two molecules. While compared vertically, both the monomer and dimer exhibited a decreased trend in the S_0_-S_1_ energy gap, and the decrease for the dimer was more significant (0.037 eV vs. 0.020 eV), which means the water could effectively reduce the S_0_-S_1_ energy gap of MAC, especially in an aggregated state. This was in accordance with the experiment result that the UV−VIS maximum absorption wavelength (measured at 1.0 × 10^−4^ mol L^−1^, isolated state) redshifted (Δ*λ* = 10 nm) with the increased solvent polarity, and $\lambda_{em}$ (measured at 5.0 × 10^−2^ mol L^−1^, aggregated state) redshifted more significantly (Δ*λ* = 38 nm). In other words, a solvent with a higher polarity can promote the TSC of MAC in solutions. Furthermore, the experimental results in which the MAC ethanol solution had a shorter $\lambda_{em}^{max}$ and lower QY, and exhibited more obvious excitation-dependent emission (EDE) than MAC crystal, can be explained as follows. The MAC in ethanol had a lower extent of aggregation than the MAC crystal, and thus a smaller TSC was formed. MAC molecules in the crystal were well-arranged and formed TSC uniformly, while in the solution, MAC molecules aggregated irregularly to form clusters with a different extent of TSC, which exhibited different emission wavelengths.

## 3. Materials and Methods

### 3.1. Materials

Hexane (AR), ethyl acetate (AR), *N*,*N*-dimethylformamide (DMF, AR), 1-butanol (AR), ethanol (AR), methanol (AR), petroleum ether (AR), and dichloromethane (AR) were purchased from Beijing Tongguang Fine Chemical Co., Ltd. (Beijing, China). Methyl acetoacetate (99%), montmorillonite clay K10 (surface area 240 m^2^/g), pyrrolidine (99%), dimethyl sulfoxide (DMSO, 99.8%), and chloroform-*d* (CDCl_3_, 99.8 atom% D, with 0.03 vol.% TMS) were purchased from InnoChem Technology Co., Ltd. (Beijing, China). Methyl 3-aminocrotonate (MAC, >97%) was purchased from TCI Development Co., Ltd. (Shanghai, China). They were used without any purification if not otherwise stated.

### 3.2. Preparation of MAC and MPB

#### 3.2.1. MAC

The MAC purchased was a yellow solid. It was first purified by column chromatography on silica gel with petroleum ether/ethyl acetate (4:1), and then recrystallized with ethyl acetate to afford a white crystal.

#### 3.2.2. MPB

The procedure was adapted from the literature [39]. Methyl acetoacetate (21.5 mL, 0.2 mol) was absorbed over montmorillonite clay K10 (50 g), then pyrrolidine (16.5 mL, 0.2 mol) was added. The sluggish mixture was stirred until dry and then left overnight. The crude product was washed from the clay with dichloromethane and then evaporated to give a yellow solid. The yellow solid was washed with water to afford a white solid. Then, it was recrystallized with ethyl acetate to obtain a white crystal (5.3 g, yield: 15.7%). The single crystal of MPB was obtained by slow volatilization in the ethyl acetate solution at room temperature.

### 3.3. Characterization

The ^1^H nuclear magnetic resonance (NMR) spectra were recorded on a JEOL-600 spectrometer (Tokyo, Japan) at room temperature, using CDCl_3_ as the solvent. The ^13^C NMR spectra were recorded on a JEOL-400 spectrometer (Tokyo, Japan) at room temperature, using CDCl_3_ as the solvent. UV−visible (UV–VIS) spectra of the solutions were measured with a UV-2600 UV−VIS spectrophotometer (Shimadzu, Kyoto, Japan) at room temperature. Single crystal X-ray diffraction data of MPB were collected on a SuperNova diffractometer (Rigaku, Tokyo, Japan) with Mo Kα radiation (*λ* = 0.71073 Å).

### 3.4. Photoluminescence Spectroscopy

The photoluminescence spectra and lifetimes of the solids were measured with an FLS-980 fluorescence spectrometer (Edinburgh instruments, Livingston, UK) at room temperature. The excitation and the emission slit widths were 2 nm. The photoluminescence spectra of the solutions were measured with an FS-5 fluorescence spectrometer (Edinburgh instruments) at room temperature. The excitation and the emission slit widths were 5 nm. The photoluminescence quantum yields were measured with a Quantaurus-QY absolute photoluminescence quantum yield spectrometer (Hamamatsu Photonics, Hamamatsu, Japan) at room temperature. Photographs of the solid powders under natural light and UV light were taken with a Mi 10 Ultra smartphone (Xiaomi, Beijing, China). Parameters for photos taken under natural light: f/1.85, 1/50 s, ISO 1198, and 6.78 mm (equivalent focal length 24 mm). The parameters for the photos taken under UV light: f/1.85, 1/9 s, ISO 339, and 6.78 mm (equivalent focal length 24 mm).

### 3.5. Computational Methods

The conformational optimization and energy calculations of the ground state were studied using density functional theory (DFT), while the first excited state was performed using time-dependent density functional theory (TD-DFT) [40] with the help of Gaussian 09 software (version D.01) [41]. PBE0 [35] and 6-311G** [36,37,38] were used as the functional and basis set for the calculations, with D3 dispersion correction [42]. The geometry optimization of MAC in water and hexane solutions was simulated using the polarizable continuum model (PCM) [43]. The absence of imaginary frequencies was carefully checked in all conformation optimizations. Atoms in molecules (AIM) analysis and electrostatic potential isosurfaces were achieved using the wave function analysis software Multiwfn (3.8, Tian Lu, Beijing, China, 2022) [44] in combination with the VMD visualization program (1.9.4, University of Illinois at Urbana-Champaign, Urbana, IL, USA, 2022) [45]. Molecular conformations and electron density distributions were plotted using IQmol software (www.iqmol.org; 2.15.3, Andrew Gilbert, Pleasanton, IL, USA, 2021).

## 4. Conclusions

In this work, the photoluminescence behaviors of two *β*-enamino esters with similar chemical structures, methyl 3-aminocrotonate and methyl (*E*)-3-(1-pyrrolidinyl)-2-butenoate, and the interactions in their aggregated structures were comparatively studied. The MAC crystal emitted a blue fluorescence under UV irradiation. MAC showed concentration-dependent and excitation-dependent fluorescence emissions. The critical cluster concentration of MAC in ethanol solutions was determined by studying the relationship between the photoluminescence intensity (UV−visible absorbance) and concentration. Below the CCC, clusters were not completely formed, and above the CCC, clusters were formed and their number increased with the increased concentration. Moreover, MAC exhibited solvatochromism, and its emission wavelength redshifted as the solvent polarity increased. On the contrary, MPB was non-emissive in both a solid state and solutions. Crystal structures and theoretical calculations proved that the H−N−C=C−C=O groups in MAC were in a planar conformation, and strong inter- and intramolecular hydrogen bonds led to the formation of large extents of TSC of MAC molecules in aggregated states. Hydrogen bonding contributes to the emission of MAC in that it acts as a bridge for electron delocalization and stiffens the conformation, and thus large TSC is formed and nonradiative decay is suppressed. No hydrogen bonds and hence no effective TSC can be formed between or within MPB molecules, and this is the reason for its non-emissive nature. This work provides a deeper understanding of how hydrogen bonding contributes to the luminescence of NTLs. It will guide the design of NTLs with a better luminescence performance.

## Figures and Tables

**Table 1:** S_0_-S_1_ energy gap of the MAC monomer and dimer in hexane and water.

Solvent	S_0_-S_1_ of Monomer/eV	S_0_-S_1_ of Dimer/eV
Hexane	5.179	4.514
Water	5.159	4.477

## Data Availability

Not applicable.

## Supplementary Materials

The following supporting information can be downloaded at: https://www.mdpi.com/article/10.3390/molecules28165950/s1. Figure S1: ^1^H NMR and ^13^C NMR spectra of MAC. Figure S2: ^1^H NMR and ^13^C NMR spectra of MPB. Figure S3: lifetime of MAC crystal under the excitation wavelength of 371.8 nm. Figure S4: UV−VIS absorption spectra of MAC ethanol solutions with different concentrations. Figure S5: photoluminescent measurement result of MPB ethanol solution (*C* = 5.0 × 10^−2^ mol L^−1^) and ethanol as the blank control under 365 nm irradiation. Figure S6: UV−VIS absorption spectra of MPB ethanol solutions with different concentrations. Figure S7: fluorescence spectra of MAC solutions, *C* = 5.0 × 10^−2^ mol L^−1^. Figure S8: UV−VIS absorption spectra of MAC solutions with different concentrations. Figure S9: optimized S_0_ conformation of MAC monomer and dimer in hexane and water.

## Sample Availability

Samples of the compounds are available from the authors.
