# Supplementary material for: Hydrogen-Bonding-Driven Nontraditional Photoluminescence of a β-Enamino Ester
Source: Molecules. 2023 Aug 8;28(16):5950. doi: 10.3390/molecules28165950 (PMC10458074; doi:10.3390/molecules28165950)
Supplement: Supplementary file 1 [file molecules-28-05950-s001.zip › molecules-2502104-supplementary.pdf]

Supplementary Materials

Hydrogen-Bonding-Driven Nontraditional Photoluminescence of a *β*-Enamino Ester

Wendi Xie [†], Junwen Deng [†], Yunhao Bai, Jinsheng Xiao and Huiliang Wang *

Beijing Key Laboratory of Energy Conversion and Storage Materials, College of Chemistry, Beijing Normal University, Beijing 100875, China

* Correspondence: wanghl@bnu.edu.cn.

† These authors contributed equally to this work.

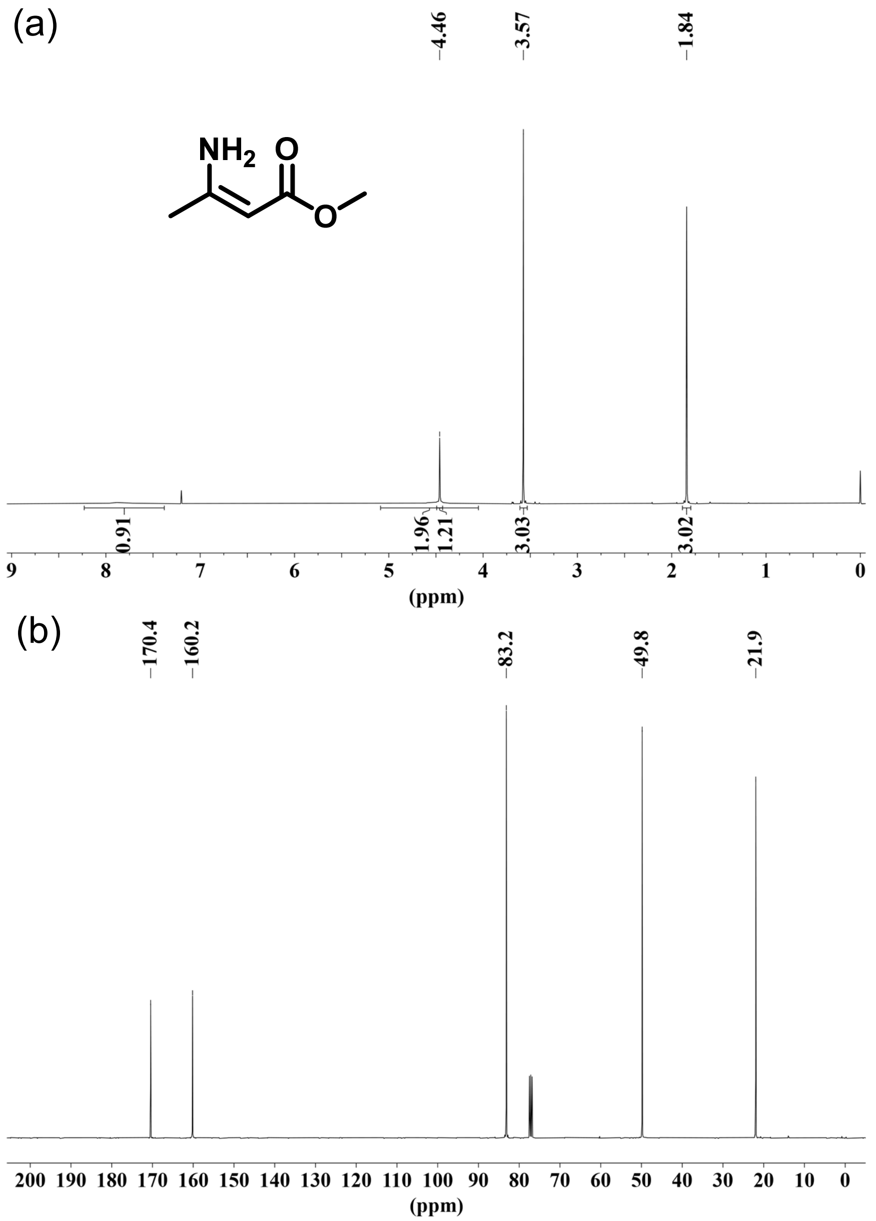

Figure S1. ^{1}H NMR (a) and ^{13}C NMR (b) spectra of methyl 3-aminocrotonate (MAC). ^{1}H NMR (600 MHz, $CDCl_3$) δ 1.84 (s, 3H), 3.57 (s, 3H), 4.46 (s, 1H), 4.1-5.2 (br, 1H), 7.7-8.5 (s, 1H); ^{13}C NMR (400 MHz, $CDCl_3$) δ 21.9, 49.8, 83.2, 160.2, 170.4.

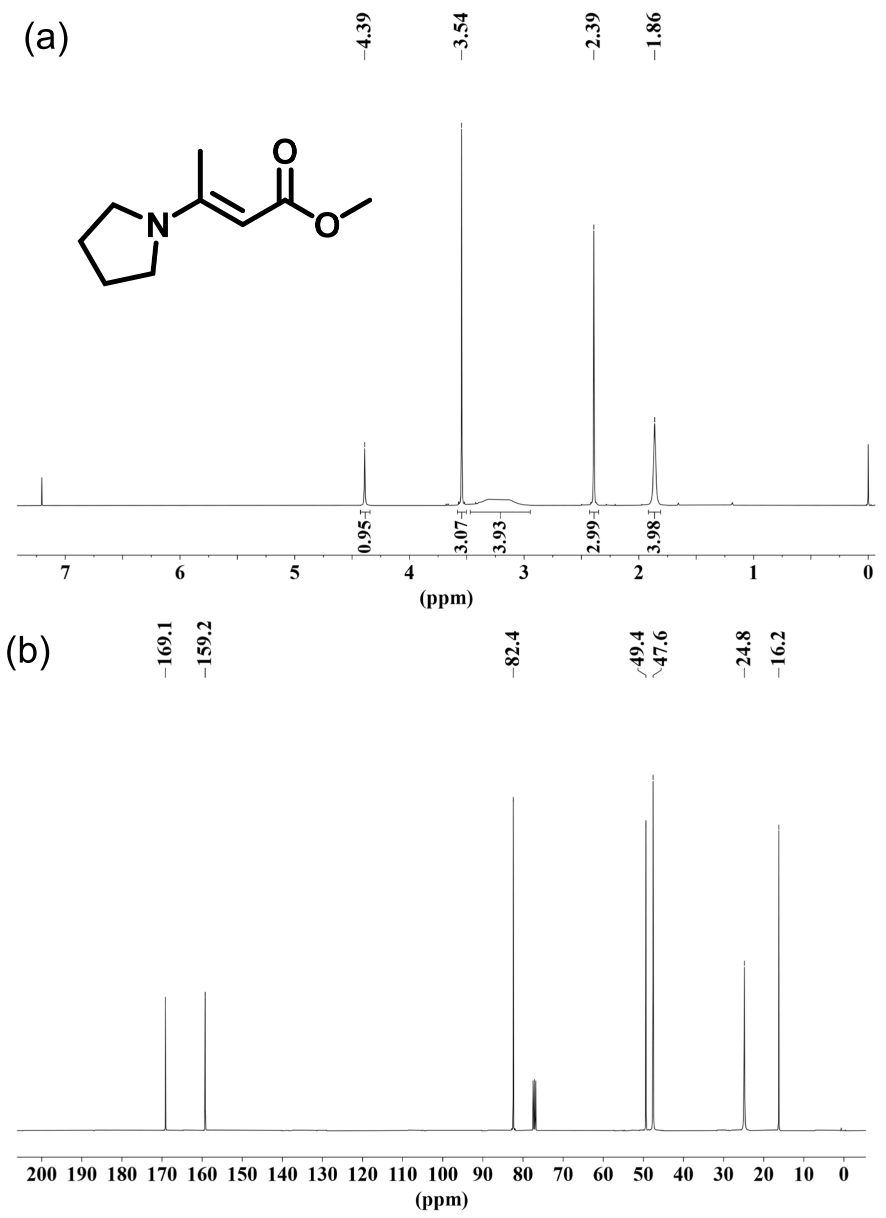

Figure S2. ^{1}H NMR (a) and ^{13}C NMR (b) spectra of methyl (*E*)-3-(1-pyrrolidinyl)-2-butenoate (MPB). ^{1}H NMR (600 MHz, $CDCl_3$) δ 1.86 (m, 4H), 2.39 (s, 3H), 2.9-3.4 (br, 4H), 3.54 (s, 3H), 4.39 (s, 1H); ^{13}C NMR (400 MHz, $CDCl_3$) δ 16.2, 24.8, 47.6, 49.4, 82.4, 159.2, 169.1.

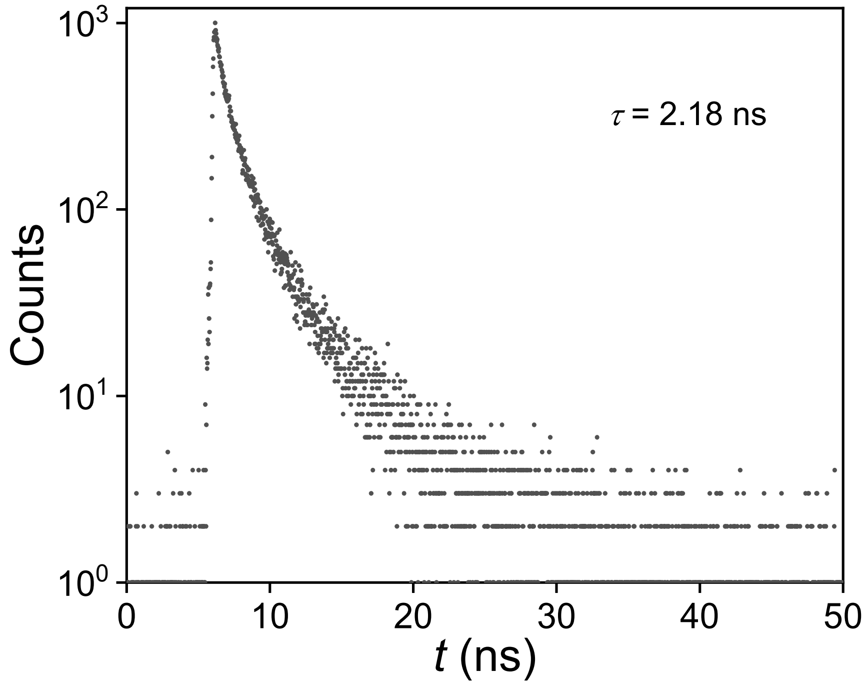

Figure S3. Lifetime of MAC crystal under the excitation wavelength of 371.8 nm.

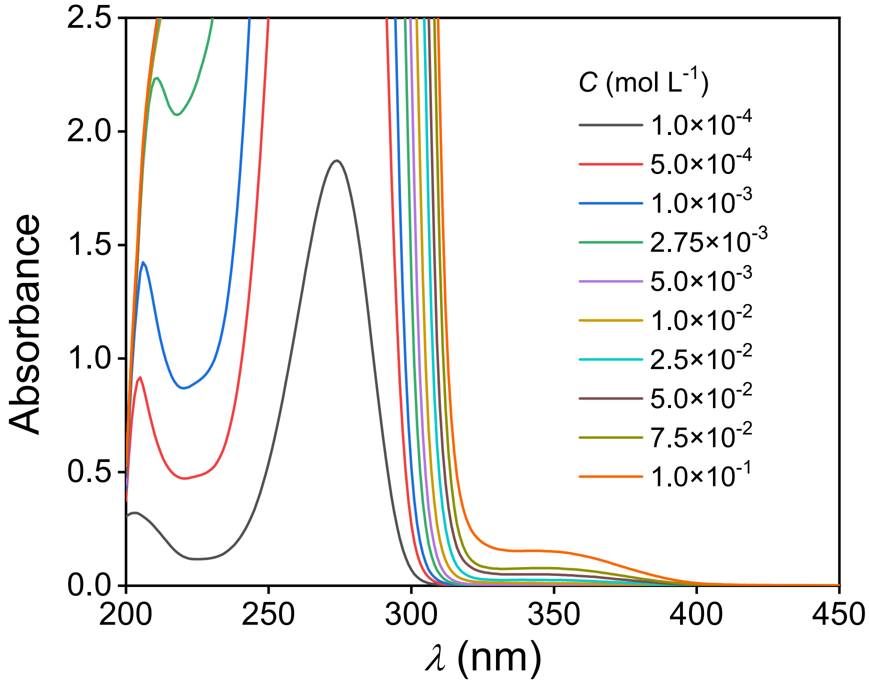

Figure S4. UV-VIS absorption spectra of MAC ethanol solutions with different concentrations.

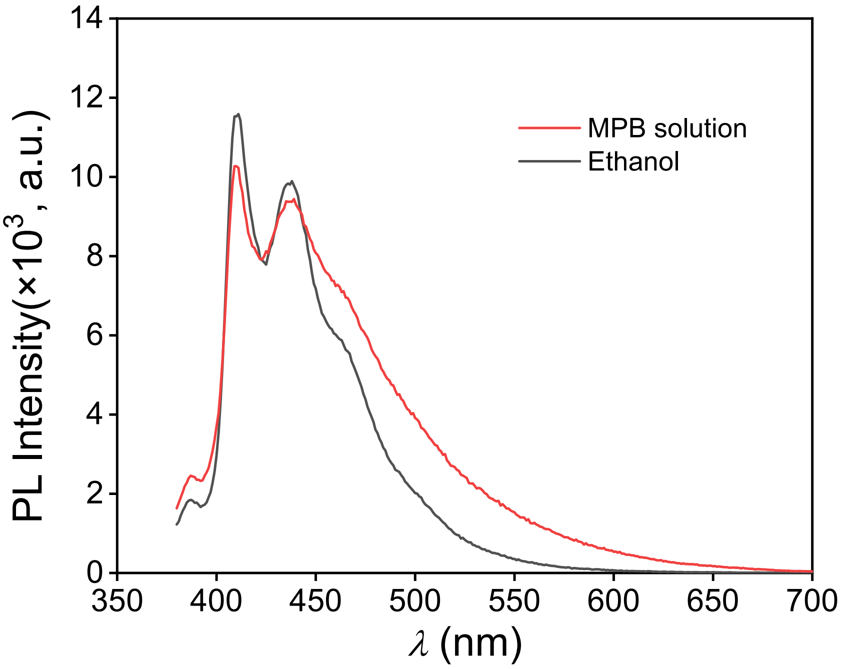

Figure S5. Photoluminescent measurement result of MPB ethanol solution (C = 5.0×10^{-2} mol L^{-1}) and ethanol as the blank control under 365 nm irradiation. The spectrum of MPB solution is not significantly different from the blank control. Therefore, MPB solution be considered as non-emissive.

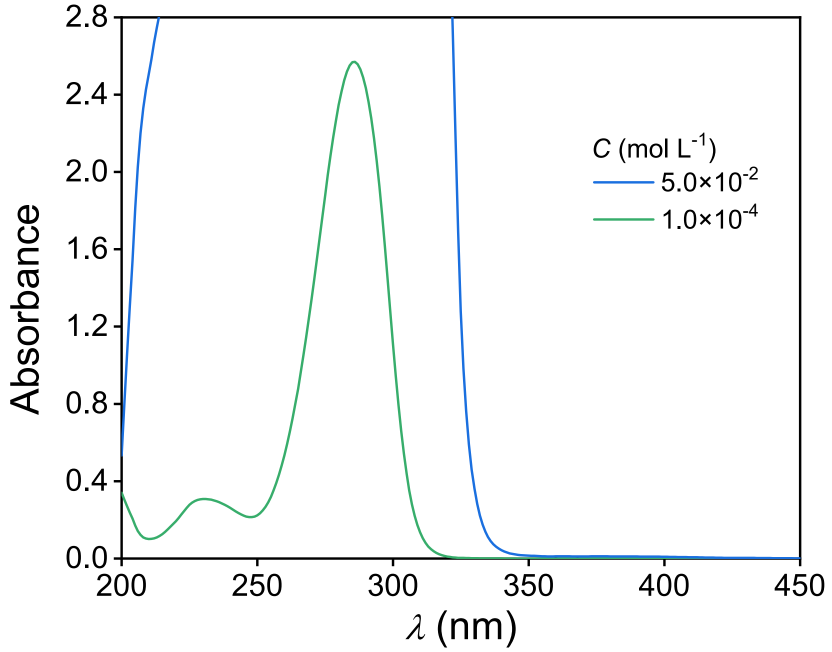

Figure S6. UV-VIS absorption spectra of MPB ethanol solutions with different concentrations.

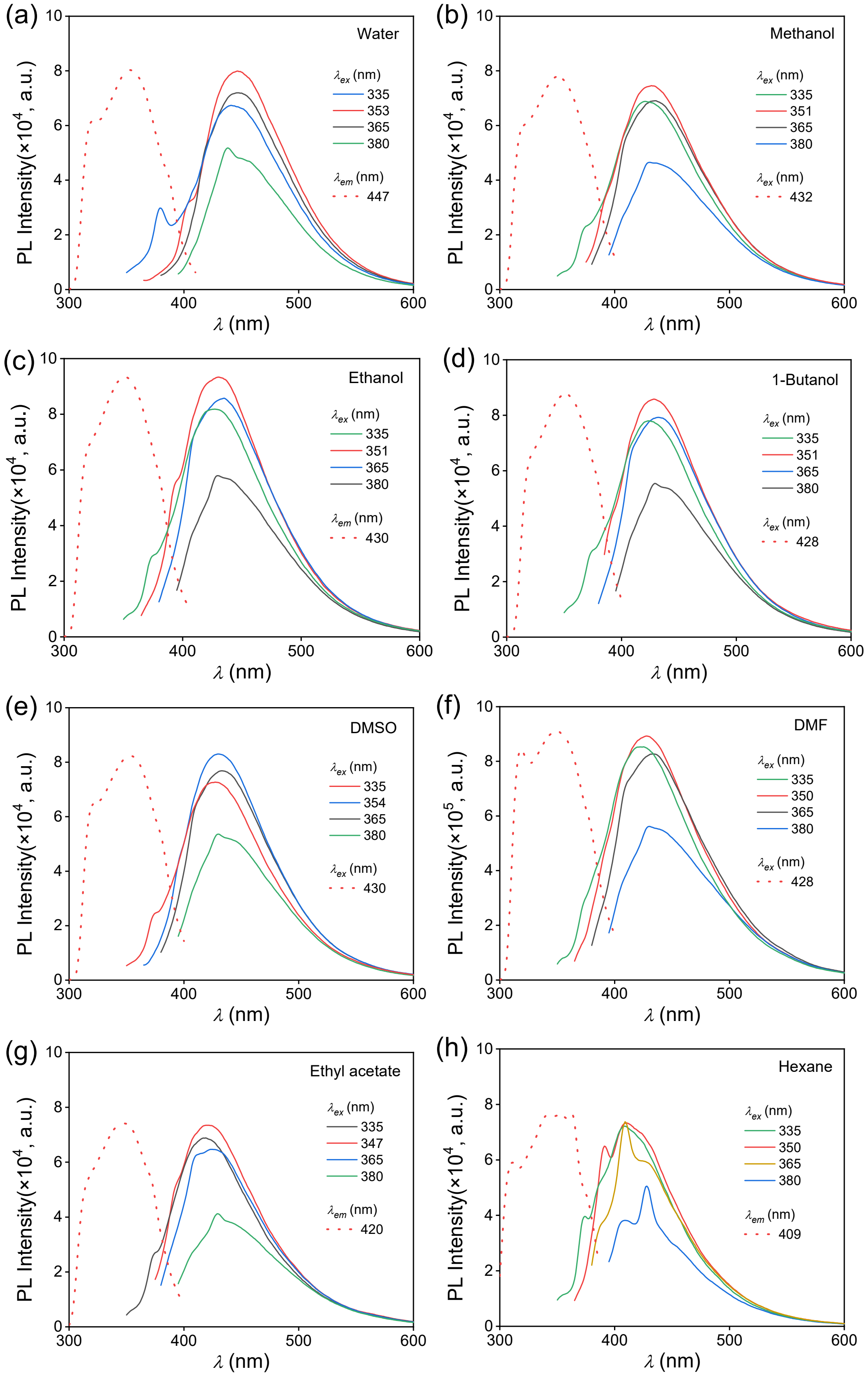

Figure S7. Fluorescence spectra of MAC solutions, $C = 5.0\times10^{-2}$ mol L^{-1}. Solvent: (a) water, (b) methanol, (c) ethanol, (d) 1-butanol, (e) DMSO, (f) DMF, (g) ethyl acetate, (h) hexane.

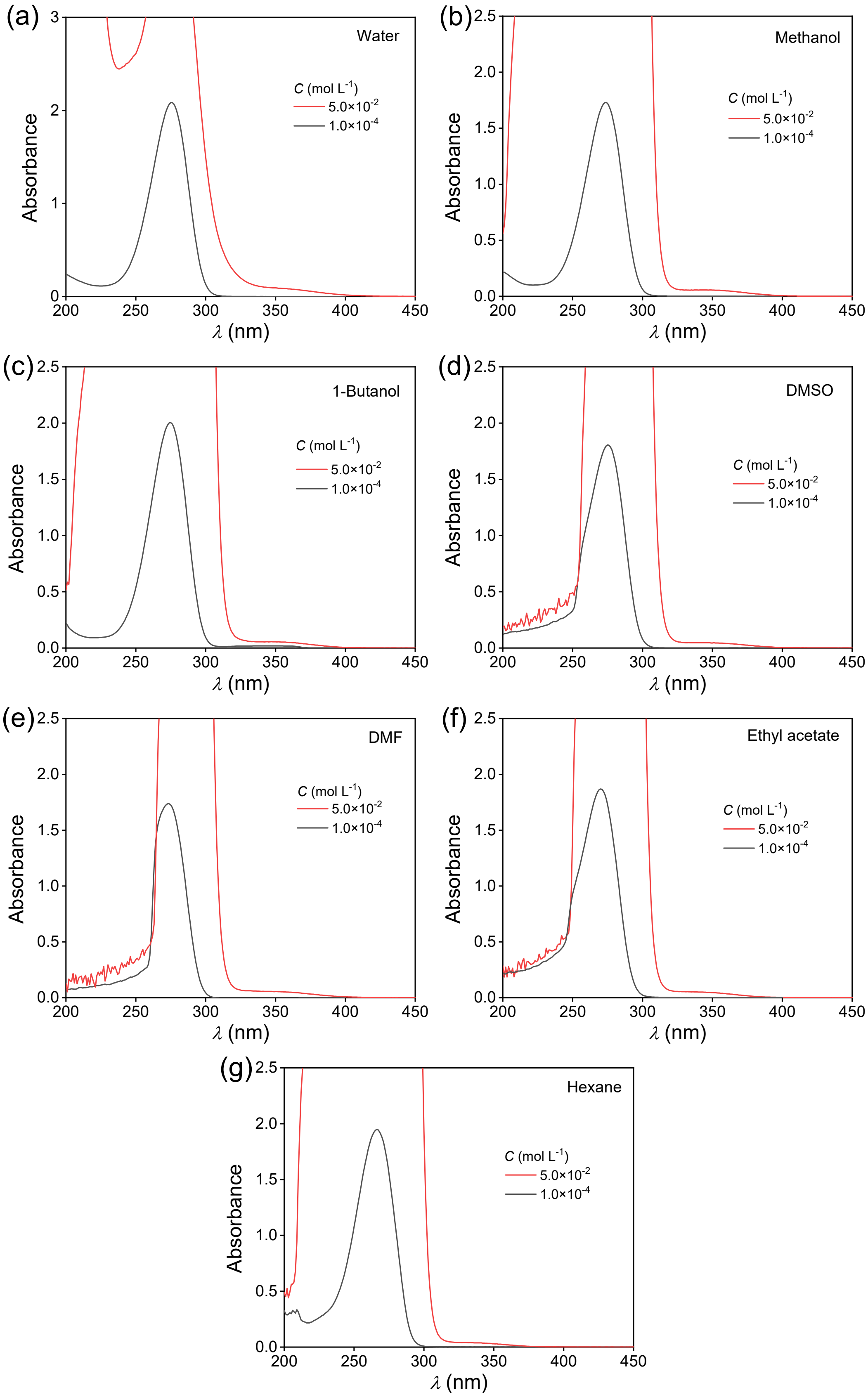

Figure S8. UV-VIS absorption spectra of MAC solutions with different concentrations. Solvent: (a) water, (b) methanol, (c) 1-butanol, (d) DMSO, (e) DMF, (f) ethyl acetate, (g) hexane.

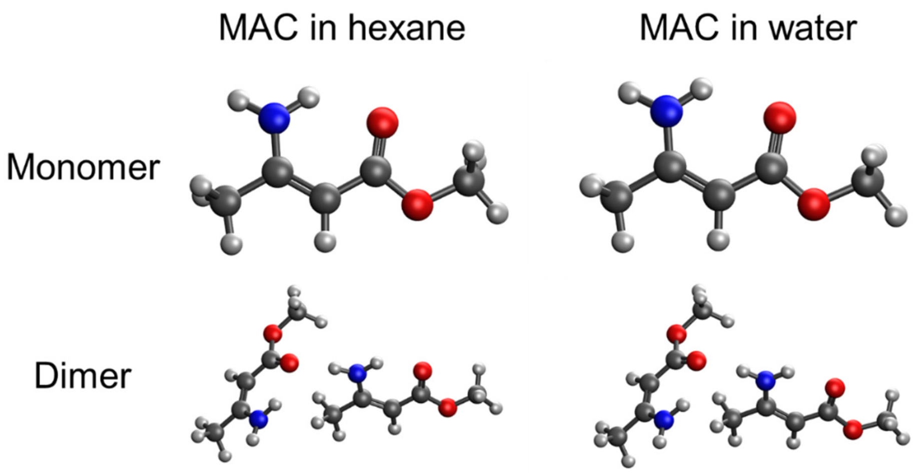

Figure S9. Optimized S_0 conformation of MAC monomer and dimer in hexane and water.

Table S1. Optimized atom coordinates of MAC monomer in hexane.

	x	y	z
O	-3.43665	-3.17965	-0.16849
O	-1.2329	-2.82367	0.07284
N	-4.19898	-5.78084	-0.20448
C	-2.3069	-3.6335	-0.03214
C	-1.94854	-5.02375	0.03567
H	-0.90601	-5.2806	0.15851
C	-2.88553	-6.01836	-0.05122
C	-2.48947	-7.45959	0.02104
H	-2.99191	-7.95019	0.8614
H	-1.41295	-7.56967	0.148
H	-2.78824	-7.9822	-0.89385
C	-1.51351	-1.4308	0.0128
H	-1.97909	-1.16283	-0.93947
H	-0.55076	-0.92981	0.11209
H	-2.17959	-1.12702	0.82487
H	-4.50542	-4.81695	-0.25812
H	-4.85646	-6.5373	-0.26497

Table S2. Optimized atom coordinates of MAC dimer in hexane.

	x	y	z
O	3.73721	0.60758	0.86586
O	4.86868	-0.83154	-0.43527
N	1.01774	0.47755	0.65381
C	3.72094	-0.29104	0.03299
C	2.55874	-0.894	-0.55485
H	2.70281	-1.68119	-1.28146
C	1.2866	-0.50326	-0.22122
C	0.09646	-1.15829	-0.85227
H	-0.57887	-1.55165	-0.08622
H	0.39766	-1.97385	-1.50953
H	-0.47252	-0.43011	-1.43929
C	6.06223	-0.2826	0.10625
H	6.14039	0.78639	-0.1105
H	6.88048	-0.8209	-0.37222
H	6.106	-0.4217	1.19006
H	1.80402	0.91046	1.12223
H	0.06378	0.67022	0.93517
O	-1.84607	0.84713	1.10947
O	-2.47118	2.14376	-0.6147
N	-3.11382	-1.53736	1.29522
C	-2.57186	1.03288	0.12938
C	-3.57704	0.13255	-0.34912
H	-4.15819	0.42676	-1.21126
C	-3.80849	-1.08426	0.23836
C	-4.85526	-2.00836	-0.29731
H	-4.40283	-2.95905	-0.59789
H	-5.36331	-1.5763	-1.15864
H	-5.60053	-2.22589	0.47498
C	-1.47947	3.08407	-0.20578
H	-1.63762	3.39467	0.82974
H	-1.59063	3.93626	-0.87489
H	-0.47582	2.66201	-0.30473
H	-2.44913	-0.91128	1.73268
H	-3.36733	-2.40203	1.74006

Table S3. Optimized atom coordinates of MAC monomer in water.

	x	y	z
O	-3.44599	-3.15791	-0.16679
O	-1.2321	-2.82211	0.07301
N	-4.21359	-5.8076	-0.21019
C	-2.3156	-3.63086	-0.03229
C	-1.96885	-5.01431	0.033
H	-0.92711	-5.2759	0.1566
C	-2.90304	-6.02188	-0.05464
C	-2.48196	-7.4532	0.0228
H	-2.97981	-7.94452	0.86455
H	-1.40346	-7.54342	0.14695
H	-2.78144	-7.98151	-0.88769
C	-1.49474	-1.41993	0.01521
H	-1.95199	-1.14682	-0.93883
H	-0.52506	-0.93444	0.11417
H	-2.15072	-1.11098	0.83256
H	-4.55469	-4.85815	-0.26759
H	-4.85277	-6.58321	-0.26636

Table S4. Optimized atom coordinates of MAC dimer in water.

	x	y	z
O	3.76653	0.50137	0.8247
O	4.77328	-0.87167	-0.65023
N	1.00225	0.51742	0.68832
C	3.66719	-0.3146	-0.09444
C	2.46188	-0.78485	-0.6958
H	2.53825	-1.50155	-1.50188
C	1.21272	-0.36215	-0.29503
C	-0.0125	-0.88802	-0.97126
H	-0.67389	-1.36022	-0.23871
H	0.24199	-1.61401	-1.74253
H	-0.5702	-0.06536	-1.42956
C	6.02332	-0.45102	-0.10405
H	6.16413	0.62501	-0.2318
H	6.78574	-0.99306	-0.66158
H	6.09097	-0.701	0.95749
H	1.79936	0.90301	1.17504
H	0.05635	0.77952	0.94395
O	-1.86821	0.93078	1.27227
O	-2.5806	2.15363	-0.47914
N	-2.95188	-1.59145	1.43916
C	-2.59742	1.03381	0.27774
C	-3.50471	0.05133	-0.21113
H	-4.09387	0.28951	-1.08573
C	-3.63738	-1.19411	0.3631
C	-4.57631	-2.19871	-0.21935
H	-4.01747	-3.0792	-0.5519
H	-5.12326	-1.78594	-1.06598
H	-5.28964	-2.53195	0.54049
C	-1.68734	3.18566	-0.05469
H	-1.92217	3.51148	0.96102
H	-1.83623	4.00719	-0.75344
H	-0.65086	2.84386	-0.09792
H	-2.29324	-0.95542	1.8671
H	-3.07298	-2.52164	1.80488

Table S5. Crystal data and structure refinement for MPB.

Identification code	2272194
Empirical formula	$C_9H_{15}NO_2$
Formula weight	169.22
Temperature/K	125(30)
Radiation	Mo Kα (λ = 0.71073)
Crystal system	triclinic
Space group	*P*-1
a/Å	6.2065(9)
b/Å	6.9681(6)
c/Å	11.3590(11)
α/°	107.299(8)
β/°	103.300(11)
γ/°	91.849(10)
Volume/Å^3	453.76(9)
Z	2
ρ_{calc} g/cm^3	1.239
Absorption coefficient/mm^{-1}	0.087
F (000)	184.0
Crystal size/mm^3	0.1 × 0.05 × 0.02
2θ range for data collection/°	6.786 to 59.814
Index ranges	$-8 \leq h \leq 8$, $-9 \leq k \leq 7$, $-15 \leq l \leq 15$
Reflections collected	3862
Independent reflections	2115 [R_{int} = 0.0428, R_{sigma} = 0.0625]
Data/restraints/parameters	2115/0/112
Goodness-of-fit on F^2	1.084
Final R indexes [I ≥2σ(I)]	R_1 = 0.0547, wR_2 = 0.1378
Final R indexes [all data]	R_1 = 0.0665, wR_2 = 0.1505
